# Floral morphology and phenology of *Sassafras tzumu* (Lauraceae)

**DOI:** 10.1186/s12870-022-03714-6

**Published:** 2022-07-08

**Authors:** Zhi Yang, Chao Tan, Yi-Min Wei, Jens G Rohwer, Bing Liu, Yong Yang

**Affiliations:** 1 Co-Innovation Center for Sustainable Forestry in Southern China, College of Biology and the Environment, Nanjing Forestry University, 159 Longpan Rd., Nanjing, 210037 China; 2 Institute of Plant Science and Microbiology, Universität Hamburg, Ohnhorststraße 18, 22609 Hamburg, Germany; 3 State Key Laboratory of Systematic and Evolutionary Botany, Institute of Botany, Chinese Academy of Sciences, 20 Nanxincun, Xiangshan, Beijing, 100093 China

**Keywords:** Flower, Lauraceae, Morphology, Phenology, *Sassafras*

## Abstract

**Background:**

*Sassafras* has been considered to belong to trib. Laureae of Lauraceae and has been assumed to have unisexual flowers. However, recent molecular phylogenetic studies have consistently suggested that *Sassafras* does not belong to the trib. Laureae but to Cinnamomeae and that it is nested within *Cinnamomum*. A recent morphological study revealed that one of the Asian species, *S. randaiense*, possesses bisexual flowers that are plesiomorphic in the family Lauraceae. As reports on the flower structure of the second Asian species, *S. tzumu*, have been contradictory, we wanted to ascertain if it has bisexual flowers or not. If the flowers were bisexual, could earlier reports that they were unisexual have been based on dichogamous flowering?

**Results:**

In this study, we investigated two populations of *S. tzumu*. We found that this species has determinate botryoid racemes, and possesses bisexual flowers. Among the three extant species, *S. tzumu* is more similar to its sister species *S. randaiense* but markedly different from the American *S. albidum*: the two Asian species possess bisexual flowers while the American species has unisexual flowers. The bisexual flower of *S. tzumu* is protogynous, and shows two phenological phases typical of Lauraceae: 1) in a flower, the pistil functions first, the stigma is fresh and white, stamens of the outer two whorls are spreading, anthers do not open, and the staminodes secrete nectar at this stage; 2) in the second phase, the stigma becomes brown, staminodes are withered, stamens of the third whorl stand up and surround the pistil, glands of the third whorl of stamens secrete nectar, and the anthers open and release pollen.

**Conclusions:**

The similarity of racemose inflorescences between *Sassafras* and some members of Laureae were caused by parallel evolution; the racemose inflorescence of ancestral *Sassafras* originated from the thyrsoid-cymose inflorescence in *Cinnamomum*. The Asian species *S. tzumu* and *S. randaiense* possess bisexual flowers with two phenological phases, the American *S. albidum* evolved unisexual flowers independently from other clades with unisexual flowers in the Lauraceae, i.e., the Laureae, *Alseodaphnopsis* in the Perseeae and the unisexual clade in the *Ocotea* complex of the Cinnamomeae.

## Introduction

*Sassafras* J. Presl is a genus of Lauraceae. Historically, the genus was considered to be related to either subtribe Cinnamomineae together with *Ocotea* Aubl., *Cinnamomum* Schaeff., *Actinodaphne* Nees, *Sassafras*, *Umbellularia* Nutt., and *Dicyellium* Nees & Mart. [1], or to trib. Laureae (other members including *Umbellularia*, *Actinodaphne*, *Dodecadenia* Nees, *Litsea* Lam., *Neolitsea* Merr., *Lindera* Thunb., *Iteadaphne* Blume, *Laurus* L., *Sassafras*, *Parasassafras* D.G. Long) [2, 3]. However, recent molecular phylogenetic studies have suggested that this genus belongs to the trib. Cinnamomeae and is closely related to *Cinnamomum* sect. *Camphora* Meisn., which causes non-monophyly of the genus *Cinnamomum* [4, 5].

*Sassafras* contains three extant species that are disjunctly distributed in East Asia and North America: *S. albidum* (Nutt.) Nees in North America, *S. randaiense* (Hayata) Rehder in Taiwan, and *S. tzumu* (Hemsl.) Hemsl. in mainland China [6]. A previous molecular dating study suggested that the divergence of the North American and Asian species took place in the late Miocene ca. 13.80–16.69 mya and the divergence between the two Asian species was around 0.61–2.23 mya. The ancestor of *Sassafras* may have lived in the thermal boreotropical flora of the high paleolatitudes of the Northern Hemisphere in the early Tertiary, and the modern disjunct distribution of *Sassafras* may have been caused by climate change from the Miocene to the late Neogene [6].

In the past, the three species of *Sassafras* have been classified in one, two, or three genera. Linnaeus [7] first recognized and described *Laurus sassafras* L. from North America. This species was considered a distinct genus by Presl [8], who consequently established *Sassafras*. Nees & Ebermaier [9] transferred *Laurus sassafras* to *Sassafras* and gave it a new name, *Sassafras officinale* T. Nees & C.H. Eberm. However, an earlier name, *Laurus albida* Nutt., is available for the North American species [10]; Nees [11] transferred it to *Sassafras*, establishing the currently accepted name *S. albidum*. Hemsley [12] described one of the Asian species under two different names in the same paper, as *Lindera tzumu* Hemsl., based on fruiting specimens, and as *Litsea laxiflora* Hemsl., based on flowering specimens (with racemose-corymbose inflorescences and 4-locular anthers). Later he placed the two species in *Sassafras* and treated *Sassafras tzumu* as the accepted name, with *Litsea laxiflora* as a synonym [13, 14]. Lecomte [15] examined flowers of *S. tzumu* and described it as having bisexual flowers, in contrast to the type species *S. albidum*. Therefore, he established a new genus, *Pseudosassafras* Lecomte, to contain only *Pseudosassafras tzumu* (Hemsl.) Lecomte. Hayata [16] described the second Asian species as *Lindera randaiensis*, based on a specimen with racemose inflorescences arranged in umbellate clusters at the tip of the branches and 2-locular anthers. Rehder [17] thought that three species were so similar in their vegetative characters (habit, bark, winter buds, deciduous leaves with a tendency toward lobing, inflorescence, and fruit) that they should be placed in a single genus. He thus classified all three species in *Sassafras*, and transferred *L. randaiensis* to *Sassafras*, as *Sassafras randaiense*. As the number of pollen sacs per anther traditionally had been considered as an important character for the separation of genera in the Lauraceae, Kamikoti [18] established a new genus, *Yushunia* Kamik., for the only species with two-locular anthers, calling it *Yushunia randaiensis* (Hayata) Kamik. Most of the other later authors, however, followed Rehder in treating the three species as a single genus [1, 2, 4, 6, 19–22], in spite of their differences in floral structure.

Different opinions were put forward on relationships of the genus *Sassafras*. Traditionally *Sassafras* was ascribed to trib. Laureae because inflorescences of the genus have been considered as racemose with an involucre or frondose transitional leaves at the base, and probably close to *Actinodaphne* [2, 3], or to subtribe Cinnamomineae of trib. Cinnamomeae because of inflorescences lacking involucral bracts, anthers 4-locular, and fruit base embedded in a cupule [1]. A detailed observation of *Sassafras randaiense* suggested that the inflorescence is a determinate botryoid raceme, and young inflorescences are enclosed within a winter bud by four to six decussate bracts [19]. Recent phylogenetic studies have suggested that *Sassafras* is sister to *Cinnamomum* sect. *Camphora* Meisn. but not to *Actinodaphne* [4, 5]. To understand the evolution of the genus better, the inflorescences of the second Asian species, *Sassafras tzumu*, are re-examined here, in order to clarify the general pattern of inflorescence structure in the genus.

Flower sex distribution is variable within in the genus. The American *Sassafras albidum* possesses unisexual flowers, which are somewhat variable in their structure. Male flowers were described as having (almost) no pistillode by Nees [11] (“Pistilli rudimentum nullum” in the genus description, “Pistilli vix ulla vestigia” in the species description), whereas van der Werff [20] mentioned that the terminal flower can have a pistillode. Female flowers were described as having six staminodes by Nuttall [10], as many stamens as in the male flowers or fewer by Nees [11], while van der Werff [20] wrote “staminodes absent or present” in the genus description and “staminodes 6” in the species description. Both Hemsley [12] and Hayata [16] considered the flowers to be unisexual when they described the Asian species; Kamikoti [18] and Keng [21] followed their opinion that the flowers of *Sassafras* are unisexual. Lecomte [15, 23] thought that *S. tzumu* possesses bisexual flowers. Gamble & Wilson [24] believed that the flowers of *S. tzumu* were polygamo-dioecious. Li et al. [22] described the genus having unisexual or bisexual flowers; they described male and female flowers separately. Chung et al. [19] observed 20 trees of *Sassafras randaiense* and finally determined that the flower of the Taiwan species is bisexual, not unisexual. It remains ambiguous whether *S. tzumu* possesses unisexual flowers, or bisexual flowers, or both.

This study is to examine the inflorescences and flowers of the Asian *Sassafras tzumu* and compare it with the American *S. albidum* and the Asian *S. randaiense* to better understand the morphological diversity and evolution of reproductive characters in the genus.

## Results

### General morphology

**Fig. 1:**
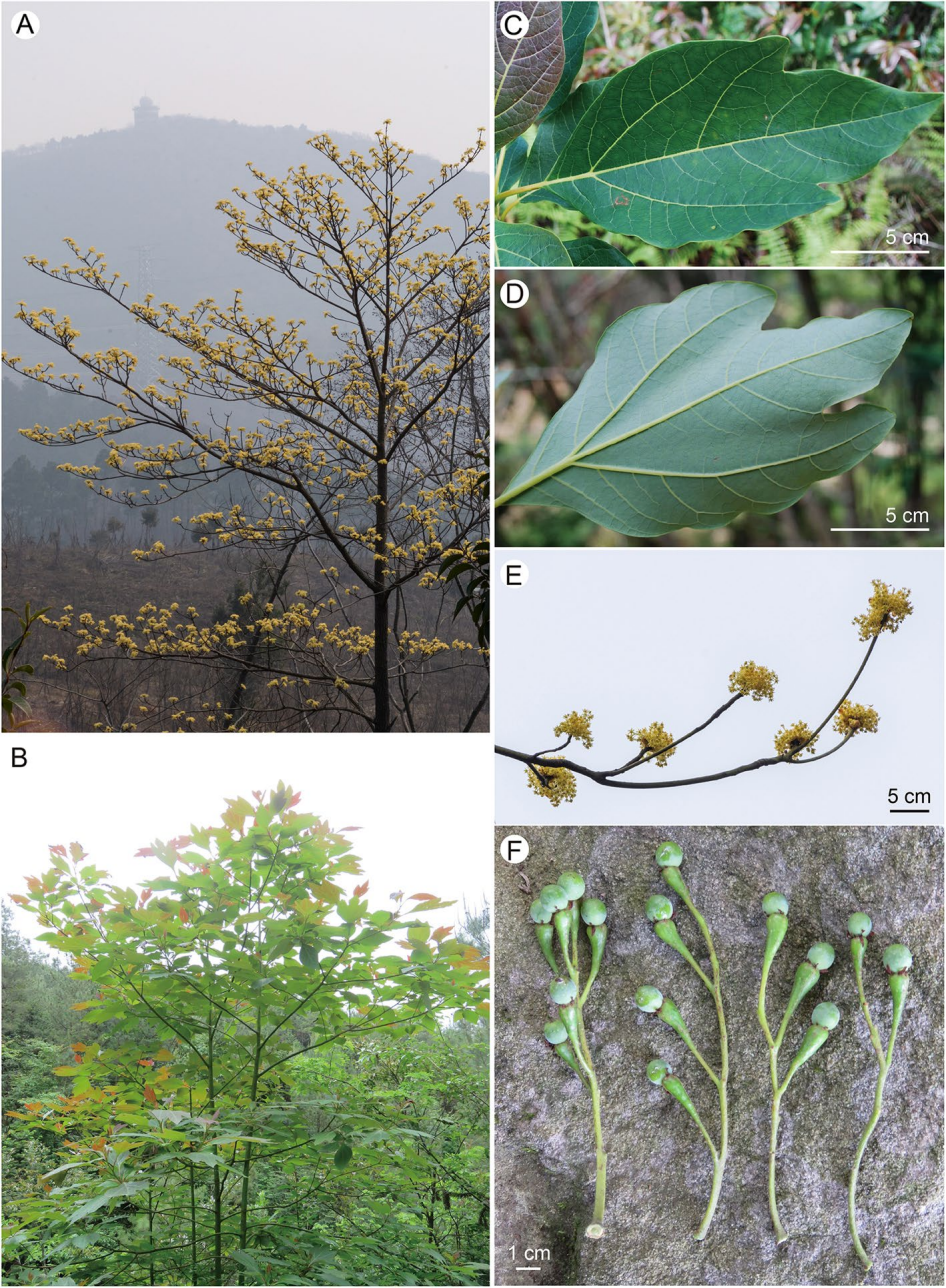
Photographs of *Sassafras tzumu* (Hemsl.) Hemsl. **A**, flowering plant in February; **B**, plant with leaves in July; **C** & **D**, leaves displaying adaxial and abaxial surfaces; **E**, flowering branch; **F**, young infructescences. Photos: **A** & **E** by Zhi Yang, **C** & **D** by Bing Liu, **B** & **F** by Feng Chen

Plants of *Sassafras tzumu* are tall deciduous trees (Fig. 1A, B). The species blooms from mid-February to early March in Nanjing (Fg. 1A). Leaves are usually trilobed and tripliveined (Fig. 1C, D). Inflorescences occur earlier than leaves. They are pseudoterminal, with a number of racemes clustered at the top of branches and subtended by a few decussate bud scales (interchangeable with ‘involucral bracts’ in Kostermans [1]) (Fig. 1E). Fruits are globose and seated on top of swollen pedicles; tepals are deciduous or rarely persistent (Fig. 1F).

### Inflorescence morphology

**Fig. 2:**
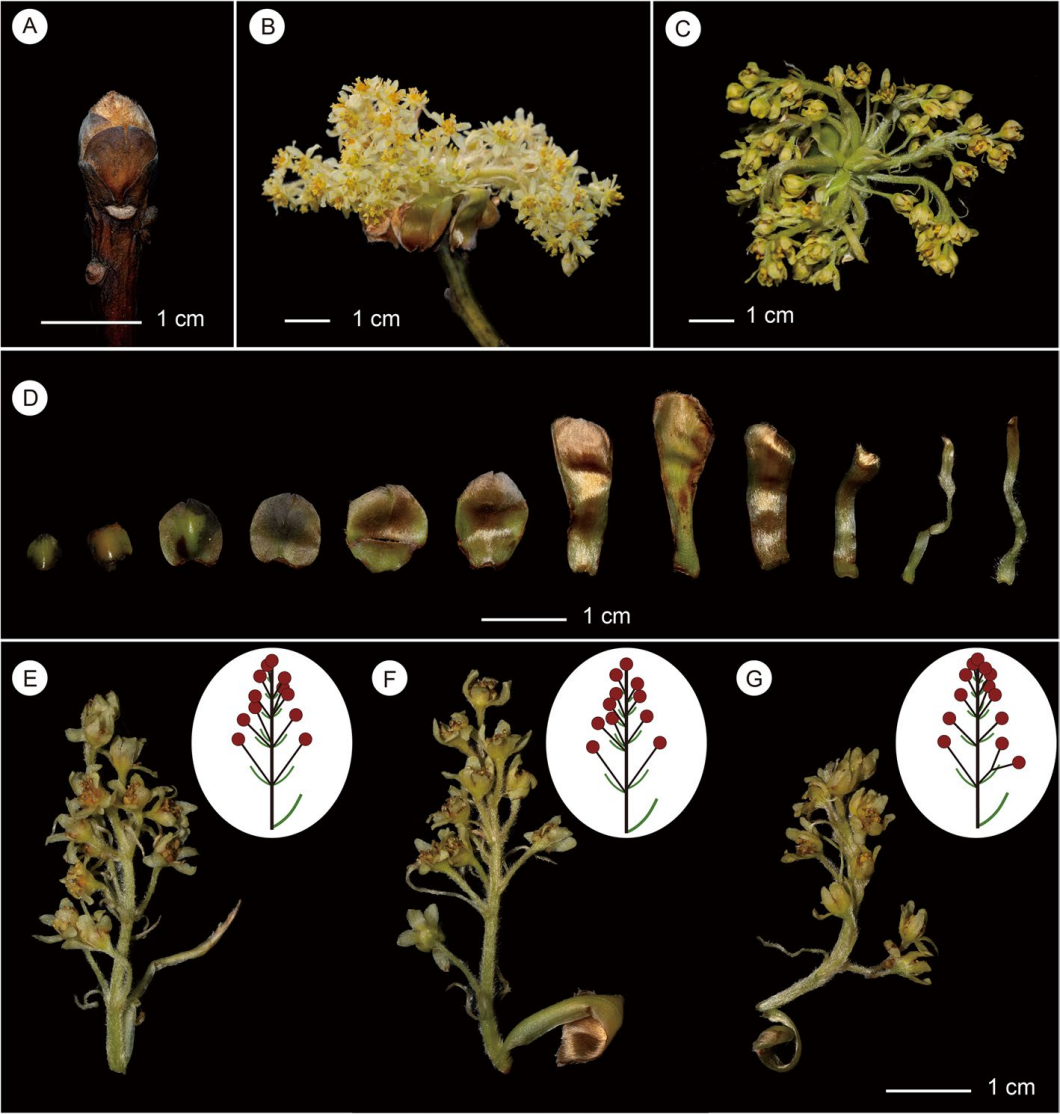
Photographs of the inflorescences of *Sassafras tzumu* (Hemsl.) Hemsl. **A**, vegetative winter bud; **B**, pseudoterminal inflorescences displaying subtending involucral bracts; **C**, pseudoterminal inflorescences displaying the true vegetative terminal bud in the center; **D**, involucral bracts displaying transitional variation from oblong bud scales to linear involucral bracts; **E**–**G**, photographs and illustrations of inflorescences displaying variation; **E**, inflorescence displaying opposite, verticillate and alternate flowers from the bottom upwards; **F**, inflorescence displaying verticillate and alternate flowers; **G**, inflorescence displaying verticillate and alternate flowers and an proximal umbel with two flowers in place of one flower. Photos by Zhi Yang

The species is deciduous and flowers occur before foliation. Inflorescences of *Sassafras tzumu* are developed from large terminal perulate buds. Buds are up to 1 cm long before they sprout; bud scales are decussate, suborbicular, and densely brownish sericeous (Fig. 2A). With the sprouting of buds, the outermost bud scales (or involucral bracts) fall off and only 4–6 more elongate inner bud scales remain (Fig. 2 B). Inflorescences are pseudoterminal; on average seven inflorescences are clustered below the true vegetative terminal buds, which will elongate and develop into normal vegetative branches (Fig. 2C). *Sassafras tzumu* has raceme-like but determinate inflorescences (= botryoids) (Figs. 2B, C & E–G). The length of the inflorescence is 4–5 cm. Involucral bracts are at the base of inflorescences and developed from bud scales, and show frondose transitional morphology from rotund to oblong and linear (Fig. 2D). In *Sassafras tzumu*, each inflorescence has about 11 pedicellate flowers. The position of the flowers on the peduncle is variable. Flowers can be arranged alternately, nearly opposite, or verticillate on the densely brownish pubescent peduncle (Fig. 2E-G). Rarely a lateral cyme with two flowers is found in place of one flower (Fig. 2G). Pedicles are 4.5–6 mm long, becoming shorter towards the tip. Bracts are linear to filiform, 1–8 mm long, distally becoming shorter.

### Flower morphology

**Fig. 3:**
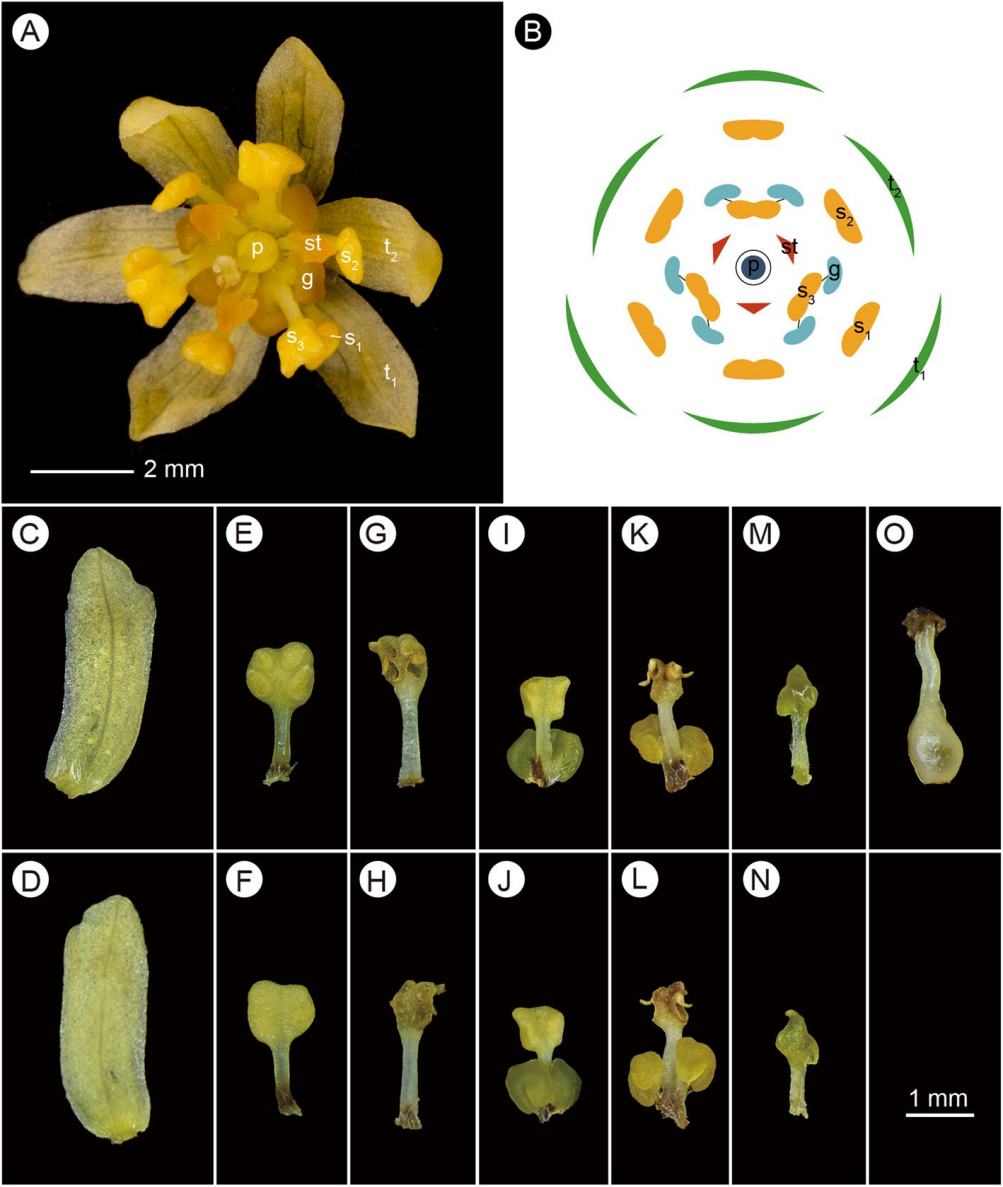
Flower morphology of *Sassafras tzumu* (Hemsl.) Hemsl. **A**, apical view of a flower; **B**, flower diagram; **C** & **D**, adaxial and abaxial surfaces of a tepal; **E** & **F**, adaxial and abaxial sides of stamens of the first and second whorls in the female phase; **G** & **H**, adaxial and abaxial sides of stamens of the first and second whorls in the male phase; **I** & **J**, adaxial and abaxial of stamens of the third whorls in the female phase; **K** & **L**, adaxial and abaxial sides of stamens of the third whorls in the male phase; **M** & **N**, adaxial and abaxial sides of a staminode; **O**, pistil. *Abbreviations*: g, glands; p, pistil; s_1_, the first whorl of stamens; s_2_, the second whorl of stamens; s_3_, the third whorl of stamens; t_1_, the first whorl of tepals; st, staminode; t_2_, the second whorl of tepals. Photos by Zhi Yang & Y.M.Wei

Flowers of *S. tzumu* are obviously bisexual, 7–9 mm in diameter. From the outside inward, the flower consists of six tepals in two whorls, nine fertile stamens in three whorls, one whorl of staminodes and a central fertile pistil (Fig. 3A, B). Tepals are yellow, lanceolate, slightly obtuse, glabrous, subequal, ca. 3 mm long (Fig. 3C, D). Fertile stamens are inserted on the rim of a very small perianth tube or receptacle; they are subequal, ca. 3 mm long. Anthers are ovoid-oblong, obtuse and emarginate at the apex. Filaments are filiform, longer than anthers, complanate, glabrous (Fig. 3E-L). Stamens of the first and second whorls are eglandular; stamens of the third whorl have a pair of glands at the base of the filaments. Glands are nearly spherical, shortly stipitate (Fig. 3I-L). Anthers of all observed flowers are 4-locular, with the upper two locules smaller than the lower two locules. All locules of the first and second whorls are ovoid-oblong, dehiscing introrsely (Fig. 3E-H). Lower locules of the third whorl are dehiscing laterally (laterorse); the upper locules are circular, dehiscing apically or slightly introrsely (Fig. 3I-L). Three staminodes alternate with stamens of the third whorl. They are ca. 1.5 mm long, glabrous, with a distinct filament and a ± triangular to somewhat caudate glandular head (Fig. 3M, N). The ovary is ovoid, ca. 1 mm long, superior, positioned in a shallow receptacle. The style is slender, ca. 1.2 mm long. The stigma is white and discoid-dilated (Fig. 3O).

### Flower phenology

**Fig. 4:**
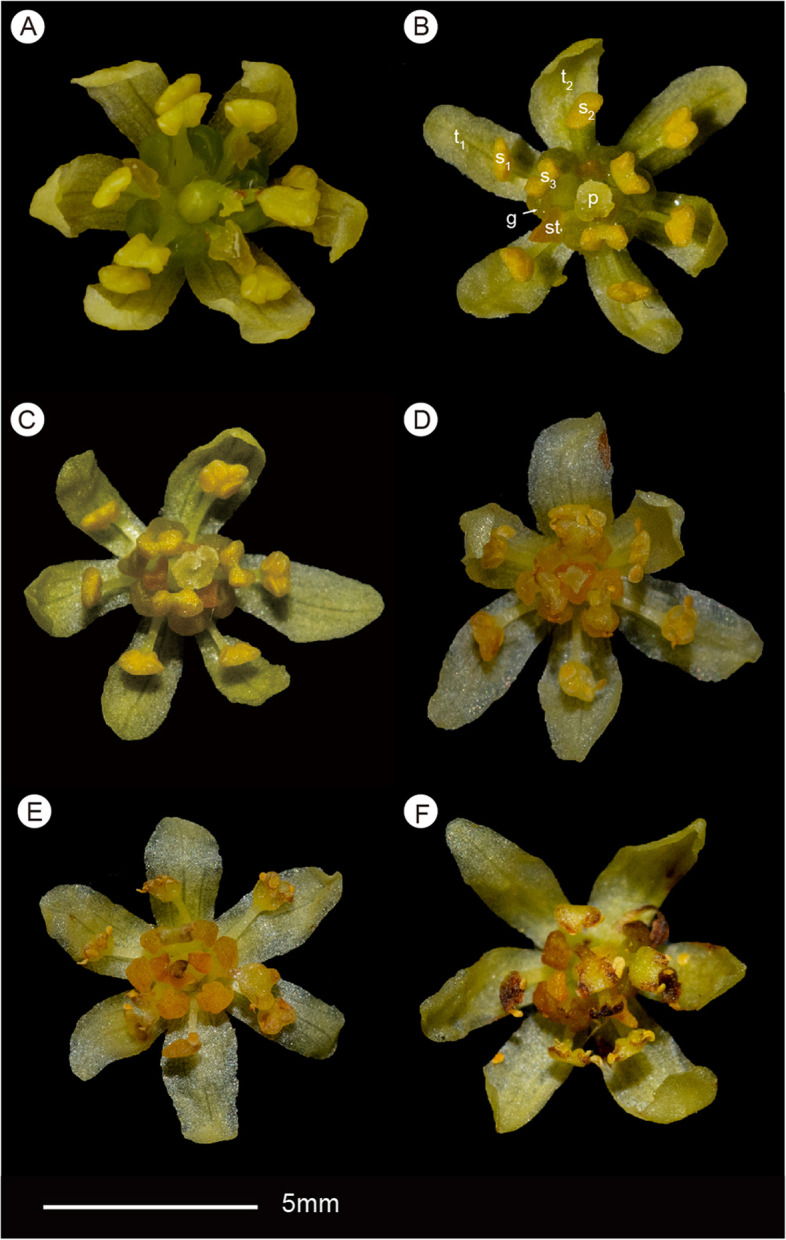
Phenology and morphological changes of flowers of *Sassafras tzumu* (Hemsl.) Hemsl. **A**, female phase, a newly opened flower displaying closed anthers, green fresh glands, and green staminodes secreting nectar; **B**, female phase, staminodes turn red, stamens of the third whorl bending inwards, staminal glands secreting nectar; **C**, female phase, staminodes and stamens of the third whorl enclosing the central pistil, stamens of the two outer whorls curving upwards; **D**, male phase, anther locules open; **E**, glands turn red at the end of pollination; **F**, flower withered. *Abbreviations*: g, glands; p, pistil; s_1_, the first whorl of stamens; s_2_, the second whorl of stamens; s_3_, the third whorl of stamens; st, staminode; t_1_, the first whorl of tepals; t_2_, the second whorl of tepals. Photos by Zhi Yang

The bisexual flower of *Sassafras tzumu* is protogynous. A newly opened flower is functionally female. In this phase, all tepals and stamens are horizontally spreading; stamens are appressed to tepals, and anther locules are closed; the glands of stamens of the third staminal whorl are green; staminodes of the forth whorl become yellow and begin to secrete nectar to attract pollinators; the stigma is white and receptive (Fig. 4A). In the male phase, the stigma becomes brownish; the staminodes turn orange and stop secreting; staminodes and stamens of the third whorl bend inward gradually, become upright and enclose the central pistil; staminal glands begin to secrete nectar (Fig. 4B); when staminodes and the third whorl of stamens enclose the pistil, the stamens of the two outer whorls also curve upwards more or less (Fig. 4C); at this time, anther locules open and the pollen is exposed to pollinators (Fig. 4D). Finally, staminal glands stop secreting and turn orange-brown, filaments are relaxed and all stamens curved inwards, the valves of the anther locules become brownish, stamens and style are withered (Fig. 4E, F).

## Discussion

Inflorescence types provide important morphological characters for the taxonomy of Lauraceae, especially at suprageneric levels [1–3]. According to van der Werff & Richter [3], there are three major types of inflorescences, thyrsoid-paniculate inflorescences lacking an involucre and possessing ultimate cymes having strictly opposite lateral flowers in the trib. Perseeae, thyrsoid-paniculate inflorescences with ultimate cymes having alternate lateral flowers in the trib. Cryptocaryeae, and racemose-umbellate inflorescences in the trib. Laureae. *Sassafras* possesses botryoid inflorescences (determinate racemes), which originate from axils of scales under a vegetative bud [2] or the axils of leaf organs transitional between bud scales and normal foliage leaves. The genus was classified into the trib. Laureae because its inflorescences were thought to belong to the same type of inflorescences as in other members of the tribe, i.e., *Actinodaphne*, *Laurus*, *Lindera*, *Litsea* etc. Recent phylogenetic studies have consistently suggested that *Sassafras* belongs to the trib. Cinnamomeae and is nested within *Cinnamomum*, while the members of the trib. Laureae form a separate clade [4, 5, 25]. As a result, it must be assumed that the racemose inflorescences of *Sassafras* and some members of the trib. Laureae (e.g. *Actinodaphne henryi* Gamble and *A. pilosa* (Lour.) Merr.) resulted from parallel evolution, and should not be considered as synapomorphic. The inflorescence of *Sassafras* is different from those of *Lindera* and *Litsea*: 1) the inflorescence of *Sassafras* is botryoid while the inflorescences of *Lindera* and *Litsea* are usually umbellate; 2) the inflorescences of *Sassafras* originate from the axils of frondose bracts below the terminal bud while the inflorescences of *Lindera* and *Litsea* are most commonly inserted on shortened branchlets [1, 2]. These differences may corroborate the parallel evolution of similar racemose inflorescences between *Sassafras* and the trib. Laureae. Kostermans [1] suggested that the involucral bracts (bud scales) of *Sassafras* are deciduous and drop before anthesis while those of *Lindera* and *Litsea* drop after anthesis. However, persistence of involucral bracts is variable in all these genera. We observed that *Lindera angustifolia* W.C. Cheng, *L. chienii* W.C. Cheng, *L. erythrocarpa* Makino, *L. megaphylla* Hemsl., *L. rubronervia* Gamble, *L. setchuenensis* Gamble possess persistent involucral bracts at anthesis whereas *L. aggregata* (Sims) Kosterm., *L. communis* Hemsl., *L. praecox* (Siebold & Zucc.) Blume, and *L. reflexa* Hemsl. have caducous involucral bracts at anthesis (pers. observ. ZY & YY). In *Sassafras albidum*, the bracts or transitional leaves subtending the inflorescences are persistent at anthesis.

**Fig. 5:**
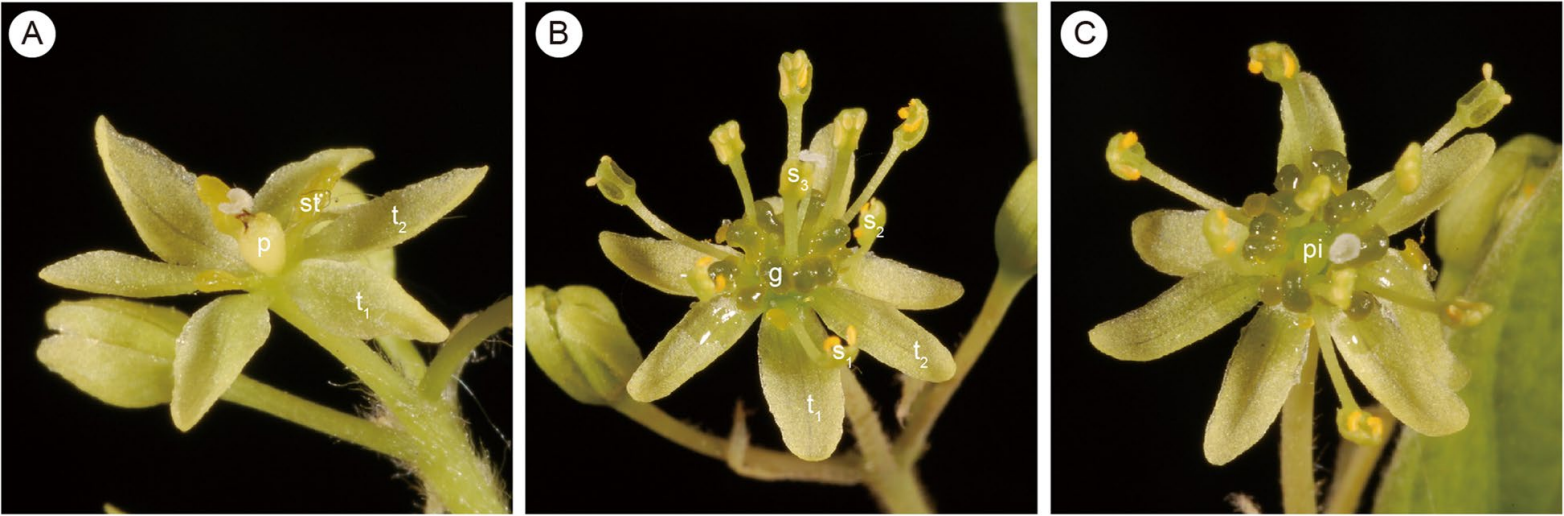
Flowers of *Sassafras albidum* (Nutt.) Nees. **A**, female flower, with three staminodes and the central functional pistil; **B**, male flower side view displaying the fertile stamens with secretory glands and staminodes of the fourth staminal whorl; **C**, male flower apical view displaying stamens, staminodes, and the central pistillode. *Abbreviations*: g, glands; p, pistil; pi, pistillode; s_1_, the first whorl of stamens; s_2_, the second whorl of stamens; s_3_, the third whorl of stamens; st, staminode; t_1_, the first whorl of tepals; t_2_, the second whorl of tepals. Photos by J.G.Rohwer

For over a century, flowers of *Sassafras* had generally been described as unisexual, and anthers of all stamens had been described as introrse [1, 22], though Lecomte [15] indicated that *S. tzumu* possesses bisexual flowers and Gamble & Wilson [24] thought the flowers of *S. tzumu* were polygamous. Chung et al. [19] observed the flowers of *Sassafras randaiense*, and found that the species possesses bisexual flowers and anthers of the third staminal whorl are extrorse. Our new observations in this study confirm that *S. tzumu* has bisexual flowers, too. Contrasting to the Asian species, the American *Sassafras albidum* has unisexual flowers (Fig. 5). Recent molecular phylogenetic studies have shown that *Sassafras* is monophyletic, so that there is no need to split it into two or three genera. It is nested within *Cinnamomum* and the two Asian species *S. tzumu* and *S. randaiense* constitute a clade sister to the American species *S. albidum* [4, 5]. As a result, it can be inferred that the ancestor of *Sassafras* possessed (predominantly) racemose inflorescences and bisexual flowers, and that the two Asian species retained the plesiomorphic bisexual flowers, whereas *S. albidum* acquired the autapomorphic unisexual flowers after its divergence from the Asian species. Unisexual flowers arose several times in the Lauraceae, e.g. in *Alseodaphnopsis* in the Perseeae [26], within the Cinnamomeae in the clade including the dioecious species of *Ocotea* as well as the dioecious genera *Endlicheria* and *Rhodostemonodaphne* [27], and in the common ancestor of the tribe Laureae.

*Sassafras* shows variation in number of anther locules. The American *S. albidum* mostly has 4-locular anthers [17], but occasionally some of the stamens may have 2-locular anthers (Fig. 5). Hemsley [12] described *Litsea laxiflora* (= *S. tzumu*) based on flowering specimens from Hubei (Badong Xian) of China; he ascribed the species to *Litsea* because the anthers are 4-locular. Hayata [16] described *Lindera randaiense* (≡*S. randaiense*) because he noticed that the species has 2-locular anthers. Chung et al. [19] observed variable locule numbers in *S. randaiense*, i.e., 2- and 4-locular anthers. We observed floral materials from two populations in mainland China, one in Nanjing of Jiangsu Province, and the other in Lushan Botanical Garden of Jiangxi Province. We found only 4-locular anthers and did not see any 2-locular anthers in *S. tzumu*. We thus confirm the observation of Hemsley [12] that *S. tzumu* possesses 4-locular anthers. In addition, we found that anthers of the first and second staminal whorl are introrse in *S. tzumu*, whereas opening of anther locules of the third staminal whorl is variable. The upper two locules are smaller, circular or nearly so, apical and/or slightly introrse, the lower two locules are relatively bigger, ovate to elliptic, and latrorse, not introrse or extrorse, which is different from the extrorse anthers in *S. randaiense* recorded by Chung et al. [19].

Anther locule number has been considered to be an important taxonomic character in the family Lauraceae. *Lindera* differs from *Litsea* in the 2-locular anthers (vs. 4-locular anthers in the latter). *Dehaasia* differs from *Alseodaphne* and *Nothaphoebe* in the 2-locular anther (vs. 4-locular anther in the latter two genera). Plants of the *Cryptocarya* group usually possess 2-locular anthers (4-locular in *Potoxylon* Kosterm.). However, 4-locular anthers are considered as the ancestral type [28], and 2-locular anthers in the family are known to have originated from 4-locular anthers multiple times because the genera with 2-locular anthers do not form a clade but belong to a number of separate clades. *Sassafras* belongs to the trib. Cinnamomeae that shares 4-locular anthers as the plesiomorphic condition, and within the genus *Sassafras* the number of anther locules changed from four to two in *S. randaiense* and occasionally in *S. albidum*. Based on observations in *S. albidum* (by JGR, pers. observ.), 2-locular anthers in *Sassafras* originated by reduction of the upper pair of locules. The 2-locular anthers of *Aiouea* Aubl. and a few *Ocotea* species resulted by reduction of the upper pair of locules as well [28]. Reduction of the lower pollen sacs is known to occur in *Urbanodendron* Mez. In the *Cryptocarya* group, 2-locular anthers arose by lateral fusion of collateral pairs of pollen sacs. However, it remains unclear why the species changed from 4-locular to 2-locular anthers. Parallel evolution of morphological characters is not rare in the family Lauraceae. Besides, plants of Lauraceae are mostly evergreen, but deciduous species occur in *Litsea*, *Lindera*, and *Sassafras*. The deciduous habit of *Sassafras* originated independently from that in *Litsea* and *Lindera*. In addition, a few deciduous species of *Lindera* possess trilobed leaves, e.g. *Lindera obtusiloba*. *Sassafras* has trilobed leaves too, which also originated separately from those in *Lindera*. Probably the convergent characters led the early authors to ascribe the Asian species of *Sassafras* to *Litsea* and *Lindera*.

In many Lauraceae, flowers are bisexual, e.g. in Perseeae, Cryptocaryeae, and most Cinnamomeae (Table 1). The bisexual flowers of the family are protogynous and show different phases during pollination [29–33]. In the female phase, the flower open, tepals and all stamens are spreading, the staminodes are yellowish and secrete nectar, the stigma is fresh and receptive [30, 31, 34]. In the male phase, the stigma becomes withered and the staminodes stop secreting, both stigma and staminodes become reddish brown, stamens of the third whorl stand up and surround the central pistil, the staminal glands of the third androecial whorl secrete nectar, anthers of all stamens open and release pollen [30, 31, 34]. After pollination, all stamens may come close together and surround the central pistil, and sometimes the tepals come close, too, e.g. in *Phoebe chekiangensis* C.B. Shang [35] (YY, pers. observ.), but sometimes not, e.g. in *Sassafras*. In this study, we found that the bisexual flower of *S. tzumu* performs in a similar way to the typical bisexual flower in *Cinnamomum*, *Persea* Mill., *Phoebe* Nees, and *Machilus* Nees [30, 31, 35]. The structure and phenological performance of bisexual flowers facilitate out-crossing in Lauraceae [29, 30]. The Asian *Sassafras* is basically consistent with the general phenology of other bisexual flowers. However, the apical or slightly introrse opening of anther locules of the third staminal whorl implies that self-pollination may be complementary when out-crossing fails. Further studies are necessary to illuminate how and why the unisexual flowers of *S. albidum* originated.

**Table 1:** Diversity of flowers of Lauraceae

Genus	Merosity	Sex	Staminodes	Locule number
*Actinodaphne* Nees	trimerous	unisexual	absent in male flowers but present in female flowers	4
*Aiouea* Aubl.	trimerous	bisexual	conspicuous	2 or 4
*Alseodaphne* Nees	trimerous	bisexual	well-developed, heart-shaped	4
*Anaueria* Kosterm.	trimerous	bisexual	conical to subulate	2
*Aniba* Aubl.	trimerous	bisexual	minute to absent	2
*Apollonias* Nees	trimerous	bisexual	conspicuous, sagittate, stipitate	2
*Aspidostemon* Rohwer & H.G.Richt.	trimerous	bisexual	subulate often united with the staminodes of whorl three	2
*Beilschmiedia* Nees	trimerous	bisexual	conspicuous, sagittate to absent	2 (rarely 4)
*Caryodaphnopsis* Airy Shaw	trimerous	bisexual	conspicuous, cordate to sagittate	4 (2)
*Cassytha* L.	trimerous	bisexual	conspicuous	2
*Chlorocardium* Rohwer & al.	tetramerous or irregular	unisexual	usually absent	4
*Cinnadenia* Kosterm.	tri- or dimerous	unisexual	absent in male flowers but present in female flowers	4
*Cinnamomum* Schaeff.	trimerous	bisexual	conspicuous, sagittate, stipitate	4
*Cryptocarya* R.Br. (including *Ravensara* Sonn.)	trimerous	bisexual	conspicuous, often sagittate	2
*Dahlgrenodendron* van der Merwe & van Wyk	trimerous	bisexual	conspicuous, ovate-triangular	2
*Damburneya* Raf.	trimerous	bisexual	with pubescent filament and a distinct rhomboidal glandular head	4
*Dehaasia* Blume	trimerous	bisexual	distinct to absent	2
*Dicypellium* Nees & Mart.	trimerous	bisexual	rarely present	4
*Dodecadenia* Nees	trimerous	unisexual	absent in male flowers	4
*Endiandra* R.Br. (including *Brassiodendron* C.K.Allen)	trimerous	bisexual	conspicuous to absent	2 (rarely 1)
*Endlicheria* Nees	trimerous	unisexual	usually absent	2 (rarely 4)
*Eusideroxylon* Teijsm. & Binn.	trimerous	bisexual	staminodes of whorl one and two tepaloid but smaller than the tepals, often with inconspicuous glands at the base, staminodes of whorl four subulate	4
*Gamanthera* van der Werff	trimerous	unisexual	absent	1 synandium formed by fusion of three stamens
*Hexapora* Hook.f.	trimerous	bisexual	enlarged, together with the stamens forming a massive cushion in the flower	2
*Hypodaphnis* Stapf	trimerous	bisexual	recognizable, with a sagittate glandular head, but fused to the glands of the adjacent ser. III stamens	4 in whorls I & II, 2 in whorl III
*Iteadaphne* Blume	trimerous	unisexual	subulate or absent in male flowers, present in female flowers	2
*Kubitzkia* van der Werff	trimerous	bisexual	absent (inner stamens fused)	2 or 4
*Kuloa* Trofimov & Rohwer	trimerous	bisexual	with a small but distinct glandular head	
*Laurus* L.	dimerous	bisexual	absent in male flowers but present in female flowers	2
*Licaria* Aubl.	trimerous	bisexual	rarely present subulate	2
*Lindera* Thunb.	trimerous	unisexual	absent in male flowers but present in female flowers	2
*Litsea* Lam.	trimerous	unisexual	absent in male flowers but present in female flowers	4
*Machilus* Nees	trimerous	bisexual	conspicuous, sagittate, stipitate	4
*Mespilodaphne* Nees	trimerous	bisexual	broadly conical	
*Mezilaurus* Kuntze ex Taub.	trimerous	bisexual	occasionally well-developed (3, 6, or 9)	2
*Nectandra* Rol. ex Rottb.	trimerous	bisexual	columnar or conical, often with a glandular patch on inside	4
*Neocinnamomum* H.Liu	trimerous	bisexual	with a glandular head	4
*Neolitsea* Merr.	dimerous	unisexual	absent in male flowers but present in female flowers	4
*Nothaphoebe* Blume	trimerous	bisexual	small	4
*Ocotea* Aubl.	trimerous	bisexual, polygamous, or unisexual	absent to conspicuous, clavate, never sagittate	4 (2)
*Paraia* Rohwer & al.	trimerous	bisexual	subulate	4
*Parasassafras* D.G.Long	trimerous	unisexual	absent	2
*Persea* Mill.	trimerous	bisexual	conspicuous, sagittate, stipitate	4 (2)
*Phoebe* Nees	trimerous	bisexual	conspicuous, sagittate, stipitate	4
*Phyllostemonodaphne* Kosterm.	trimerous	bisexual	rarely present, minute	2
*Pleurothyrium* Nees	trimerous	bisexual	present but hidden between the glands	4
*Potameia* Thouars	dimerous	bisexual	absent	2
*Potoxylon* Kosterm.	trimerous	bisexual	large, triangular, glandular on inside	4
*Povedodaphne* Burger	trimerous	bisexual	absent	4
*Rhodostemonodaphne* Rohwer & Kubitzki	trimerous	unisexual	absent	4
*Sassafras* L. ex Nees	trimerous	unisexual (*S. albidum*) or bisexual (*S. tzumu* & *S. randaiense*)	minute to absent	2 or 4
*Sinopora* J.Li & al.	trimerous	bisexual	present, as large as stamens	2
*Syndiclis* Hook.f.	trimerous or dimerous	bisexual	present	2 (or 1)
*Umbellularia* Nutt.	trimerous	bisexual	distinct, slightly glandular at the tip	4
*Urbanodendron* Mez	trimerous	bisexual	rarely present	2 or 4
*Williamodendron* Kubitzki & H.G.Richt.	trimerous	bisexual	subulate	4
*Yasunia* van der Werff	trimerous or dimerous	bisexual	columnar	2

*Note*: floral characters of genera of Lauraceae extracted mainly from Rohwer [2]; *Hypodaphnis* was examined by J.G.Rohwer (vouchers: Leeuwenberg 5557, Wilks 1655, Zenker 3033a in HBG); *Sinopora* is according to Li et al. [36]; *Yasunia* is from van der Werff & Nishida [37]; *Damburneya* is from Trofimov et al. [38]

Though both *Sassafras tzumu* and *S. albidum* are deciduous, the former is different from the latter in the flowering time relative to leaf development. *Sassafras tzumu* flowers earlier than leaf sprouting while *S. albidum* flowers simultaneously with the flushing of the leaves, with inflorescences originating mainly from the axils of leaf organs that are transitional between bud scales and foliage leaves (pers. observ. by J.G.Rohwer). This furthers our understanding on the divergent evolution of the two species.

## Materials and methods

Totally 19 individuals of *Sassafras tzumu* were observed and sampled in late February and early March of 2022. Three of them were collected from the Lushan Botanical Garden, Jiujiang City, Jiangxi Province, China (28° 51′ 2" N, 115° 46′ 59" E, elev. 142 m), others were collected from Shecun village, Jiangning District of Nanjing, Jiangsu Province, China (32° 0′ 33" N, 118° 56′ 22" E, elev. 118 m). Flowering branches were collected in the field; at least three reproductive shoots were included for each individual. Floral materials were fixed in FAA (mixture of Formalin, Alcohol, and Glacial Acetic Acid). Measurements and observations of flowers were conducted in the Laboratory of Systematic and Evolutionary Botany, Nanjing Forestry University. Photographs were taken using a Nikon D7100 with micro lens (AF Micro-Nikkor 60 mm F2.8D). Detailed morphological observations and photographs were also made under a Stereo Microscope (OLYMPUS SZX10). Voucher specimens of *Sassafras tzumu* (Y.Yang, Z.Yang & C.Tan QLS-1) were deposited in the Herbarium NF, Nanjing Forestry University; a specimen of *S. albidum* (Rohwer s.n., 24 Sep 2002) was deposited in the Herbarium HBG, University of Hamburg (Germany). All specimens and materials examined were identified by Yong Yang; pictures of *S. albidum* were taken in the Botanic Garden of Hamburg (Germany) and identified by Jens G. Rohwer.

## Data Availability

All data used in the study are included in this paper.
